# Supplementary material for: A Critical View of the Application of the APEX Software (Aqueous Photochemistry of Environmentally-Occurring Xenobiotics) to Predict Photoreaction Kinetics in Surface Freshwaters
Source: Molecules. 2019 Dec 18;25(1):9. doi: 10.3390/molecules25010009 (PMC7017383; doi:10.3390/molecules25010009)
Supplement: Supplementary file 1 [file molecules-25-00009-s001.zip › 3.2.4_gcc-4.4.0.zip!/3.2.4_gcc-4.4.0/3.2.4_gcc-4.4.0/doc/octave/html/interpreter/Installing-and-Removing-Packages.html]

Installing and Removing Packages - Untitled


Next: Using Packages,
Up: Packages


---

### 35.1 Installing and Removing Packages

Assuming a package is available in the file image-1.0.0.tar.gz
it can be installed from the Octave prompt with the command

```
     pkg install image-1.0.0.tar.gz
```

If the package is installed successfully nothing will be printed on
the prompt, but if an error occurred during installation it will be
reported. It is possible to install several packages at once by
writing several package files after the `pkg install` command.
If a different version of the package is already installed it will
be removed prior to installing the new package. This makes it easy to
upgrade and downgrade the version of a package, but makes it
impossible to have several versions of the same package installed at
once.

To see which packages are installed type

```
     pkg list
     -| Package Name  | Version | Installation directory
     -| --------------+---------+-----------------------
     -|        image *|   1.0.0 | /home/jwe/octave/image-1.0.0
```

In this case only version 1.0.0 of the `image` package is
installed. The '\*' character next to the package name shows that the
image package is loaded and ready for use.

It is possible to remove a package from the system using the
`pkg uninstall` command like this

```
     pkg uninstall image
```

If the package is removed successfully nothing will be printed in the
prompt, but if an error occurred it will be reported. It should be
noted that the package file used for installation is not needed for
removal, and that only the package name as reported by `pkg list`
should be used when removing a package. It is possible to remove
several packages at once by writing several package names after the
`pkg uninstall` command.

To minimize the amount of code duplication between packages it is
possible that one package depends on another one. If a package
depends on another, it will check if that package is installed
during installation. If it is not, an error will be reported and
the package will not be installed. This behavior can be disabled
by passing the `-nodeps` flag to the `pkg install`
command

```
     pkg install -nodeps my_package_with_dependencies.tar.gz
```

Since the installed package expects its dependencies to be installed
it may not function correctly. Because of this it is not recommended
to disable dependency checking.

— Command: pkg command pkg\_name  
— Command: pkg command option pkg\_name  
> This command interacts with the package manager. Different actions will
> be taken depending on the value of command.
>
> install: Install named packages. For example, ``` pkg install image-1.0.0.tar.gz ``` installs the package found in the file image-1.0.0.tar.gz. The option variable can contain options that affect the manner in which a package is installed. These options can be one or more of `-nodeps`: The package manager will disable the dependency checking. That way it is possible to install a package even if it depends on another package that's not installed on the system. **Use this option with care.** `-noauto`: The package manager will not automatically load the installed package when starting Octave, even if the package requests that it is. `-auto`: The package manager will automatically load the installed package when starting Octave, even if the package requests that it isn't. `-local`: A local installation is forced, even if the user has system privileges. `-global`: A global installation is forced, even if the user doesn't normally have system privileges `-verbose`: The package manager will print the output of all of the commands that are performed. uninstall: Uninstall named packages. For example, ``` pkg uninstall image ``` removes the `image` package from the system. If another installed package depends on the `image` package an error will be issued. The package can be uninstalled anyway by using the `-nodeps` option. load: Add named packages to the path. After loading a package it is possible to use the functions provided by the package. For example, ``` pkg load image ``` adds the `image` package to the path. It is possible to load all installed packages at once with the command ``` pkg load all ``` unload: Removes named packages from the path. After unloading a package it is no longer possible to use the functions provided by the package. This command behaves like the `load` command. list: Show a list of the currently installed packages. By requesting one or two output argument it is possible to get a list of the currently installed packages. For example, ``` installed_packages = pkg list; ``` returns a cell array containing a structure for each installed package. The command ``` [user_packages, system_packages] = pkg list ``` splits the list of installed packages into those who are installed by the current user, and those installed by the system administrator. describe: Show a short description of the named installed packages, with the option '-verbose' also list functions provided by the package, e.g.: ``` pkg describe -verbose all ``` will describe all installed packages and the functions they provide. If one output is requested a cell of structure containing the description and list of functions of each package is returned as output rather than printed on screen: ``` desc = pkg ("describe", "secs1d", "image") ``` If any of the requested packages is not installed, pkg returns an error, unless a second output is requested: ``` [ desc, flag] = pkg ("describe", "secs1d", "image") ``` flag will take one of the values "Not installed", "Loaded" or "Not loaded" for each of the named packages. prefix: Set the installation prefix directory. For example, ``` pkg prefix ~/my_octave_packages ``` sets the installation prefix to ~/my\_octave\_packages. Packages will be installed in this directory. It is possible to get the current installation prefix by requesting an output argument. For example, ``` p = pkg prefix ``` The location in which to install the architecture dependent files can be independent specified with an addition argument. For example ``` pkg prefix ~/my_octave_packages ~/my_arch_dep_pkgs ``` local\_list: Set the file in which to look for information on the locally installed packages. Locally installed packages are those that are typically available only to the current user. For example ``` pkg local_list ~/.octave_packages ``` It is possible to get the current value of local\_list with the following ``` pkg local_list ``` global\_list: Set the file in which to look for, for information on the globally installed packages. Globally installed packages are those that are typically available to all users. For example ``` pkg global_list /usr/share/octave/octave_packages ``` It is possible to get the current value of global\_list with the following ``` pkg global_list ``` rebuild: Rebuilds the package database from the installed directories. This can be used in cases where for some reason the package database is corrupted. It can also take the `-auto` and `-noauto` options to allow the autoloading state of a package to be changed. For example ``` pkg rebuild -noauto image ``` will remove the autoloading status of the image package. build: Builds a binary form of a package or packages. The binary file produced will itself be an Octave package that can be installed normally with `pkg`. The form of the command to build a binary package is ``` pkg build builddir image-1.0.0.tar.gz ... ``` where `builddir` is the name of a directory where the temporary installation will be produced and the binary packages will be found. The options `-verbose` and `-nodeps` are respected, while the other options are ignored.
